# A Mechanical Model to Interpret Cell-Scale Indentation Experiments on Plant Tissues in Terms of Cell Wall Elasticity and Turgor Pressure

**DOI:** 10.3389/fpls.2016.01351

**Published:** 2016-09-07

**Authors:** Richard Malgat, François Faure, Arezki Boudaoud

**Affiliations:** ^1^Institut National de Recherche en Informatique et en AutomatiqueGrenoble, France; ^2^Laboratoire Jean Kuntzmann, Centre National de la Recherche ScientifiqueGrenoble, France; ^3^Reproduction et Développement des Plantes, Université de Lyon, Ecole Normale Supérieure de Lyon, Université Claude Bernard Lyon 1, Institut National de la Recherche Agronomique, Centre National de la Recherche ScientifiqueLyon, France

**Keywords:** mechanical model, cell wall, turgor, physically-based simulation, atomic force microscope, indentation, shoot apical meristem, floral meristem

## Abstract

Morphogenesis in plants is directly linked to the mechanical elements of growing tissues, namely cell wall and inner cell pressure. Studies of these structural elements are now often performed using indentation methods such as atomic force microscopy. In these methods, a probe applies a force to the tissue surface at a subcellular scale and its displacement is monitored, yielding force-displacement curves that reflect tissue mechanics. However, the interpretation of these curves is challenging as they may depend not only on the cell probed, but also on neighboring cells, or even on the whole tissue. Here, we build a realistic three-dimensional model of the indentation of a flower bud using SOFA (Simulation Open Framework Architecture), in order to provide a framework for the analysis of force-displacement curves obtained experimentally. We find that the shape of indentation curves mostly depends on the ratio between cell pressure and wall modulus. Hysteresis in force-displacement curves can be accounted for by a viscoelastic behavior of the cell wall. We consider differences in elastic modulus between cell layers and we show that, according to the location of indentation and to the size of the probe, force-displacement curves are sensitive with different weights to the mechanical components of the two most external cell layers. Our results confirm most of the interpretations of previous experiments and provide a guide to future experimental work.

## 1. Introduction

Morphogenesis relies on well-defined patterns of growth determined by gene expression (Coen et al., 2004). Mechanistically, it is thought that gene activity influences the mechanical properties of tissues at cellular and sub-cellular level (Traas and Monéger, 2010; Mirabet et al., 2011; Robinson et al., 2013; Ali et al., 2014), thus controlling patterns of growth. Plants are well-suited to investigate the mechanistic basis of morphogenesis: Growth is limited by the polysaccharide-made walls that surround cells and is driven by the osmotically-generated turgor pressure, which puts walls in tension (Schopfer, 2006).

Several lines of evidence indicate links between gene expression, cell wall properties and growth, notably at the shoot apex. The activity of cell wall remodeling proteins, expansins (Fleming et al., 1997; Cho and Cosgrove, 2000; Pien et al., 2001) and pectinmethyl esterases (Peaucelle et al., 2008), enables outgrowth or enhances growth. The first steps of flower organogenesis are associated with a reduction in cell wall stiffness (Peaucelle et al., 2011; Braybrook and Peaucelle, 2013). More generally, there is an inverse correspondence between wall stiffness and growth rate (Milani et al., 2013). Finally, cell stiffness correlates with the expression of CLAVATA3, the glycopeptide associated with the central zone (Milani et al., 2014).

Here, we aim at providing a theoretical framework to interpret measurements of plant mechanics at cellular resolution. Frameworks at organ scale have a long history (Niklas, 1992) and enable the deduction of average properties of cell layers from experiments where the whole organ is stretched or bent (Niklas, 1992), based for instance on models that link cell wall elastic modulus (the higher the modulus, the stiffer the wall), turgor pressure, and average elastic modulus of the tissue (Nilsson et al., 1958).

Experimental approaches to measure mechanical properties have been recently scaled down thanks to nano-indentation systems, such as atomic force microscopes, whereby a nanometric to micrometric probe is used to apply a force in the nN-μN range on the sample of interest while its displacement is monitored, yielding force-displacement curves at well-defined locations. Measurements were performed on the shoot apical meristem of Arabidopsis (Milani et al., 2011; Peaucelle et al., 2011; Braybrook and Peaucelle, 2013; Milani et al., 2014), on roots (Fernandes et al., 2012), on cotyledons and leaves (Hayot et al., 2012; Forouzesh et al., 2013; Sampathkumar et al., 2014), on onion scales (Lintilhac et al., 2000; Routier-Kierzkowska et al., 2012; Beauzamy et al., 2015a), on pollen tubes (Geitmann and Parre, 2004; Vogler et al., 2013), or on culture cells (Radotic et al., 2012; Weber et al., 2015). These approaches (reviewed in Geitmann, 2006; Milani et al., 2013; Routier-Kierzkowska and Smith, 2013; Beauzamy et al., 2014; Vogler et al., 2015) have contributed to a renewal of plant biomechanics (Moulia, 2013).

The interpretation of nano-indentation experiments raises a number of questions. Indeed, when a piece of wall is stretched, the elastic modulus that quantifies wall stiffness can be obtained directly from the slope of force vs. displacement curve because the surface on which the force is applied remains constant (Boudaoud, 2010), whereas in indentation experiments on live tissues, the surface of contact between the probe and the sample increases with depth and, in addition, turgor pressure may contribute to the mechanical response. What information is revealed by force-displacement curves? Are measurements sensitive to the cell wall elasticity, cell wall viscosity, and/or to turgor pressure? Are measurements sensitive to the properties of the cell indented or of a larger group of cells? Do probes of different sizes reveal properties at different scales (Peaucelle et al., 2011)? These are typical questions that we tackle in the present study. To do so, we need to build realistic mechanical models of plant tissues.

Such models have been worked out at the subcellular scale. When indentation depth is small with respect to wall thickness and the wall is assumed to be locally homogeneous, standard models from contact mechanics (Johnson, 1987) enable the deduction of the transverse elastic modulus (Milani et al., 2011), which quantifies the wall stiffness in the direction of its thickness. The assumption of homogeneity can be relieved by assuming that the elastic modulus varies smoothly with the distance normal to the surface (Lee et al., 2009) or that the wall is made of two types of homogeneous materials (Roduit et al., 2009). The assumption of linear elastic behavior of the material can be relieved in models that include nonlinear elasticity (Valero et al., 2016) or viscoelastoplastic behavior (Tvergaard and Needleman, 2011). Cell-scale models can account for the cell wall and for turgor (Routier-Kierzkowska et al., 2012), leading to the deduction of wall elastic modulus (Hayot et al., 2012) or turgor pressure (Forouzesh et al., 2013) from indentation experiments. Physical models can also be combined to extract many physical parameters from a single force-displacement curve (Beauzamy et al., 2015a; Bonilla et al., 2015). Incidentally, inferring turgor provides a useful alternative to the more standard pressure probe (Tomos and Leigh, 1999), in which cells are impaled for measurements. In tissue-scale models (Hamant et al., 2008; Bassel et al., 2014; Sampathkumar et al., 2014), too much spatial detail—a resolution smaller than wall thickness—would lead to a huge computational time; therefore cell walls are modeled as thin plates or shells and wall thickness is accounted for only through stretching and bending moduli, which quantify the stiffness of the plate when stretched or bent, respectively. In such shell models, indentation has been explored only in geometries corresponding to single cells in the shape of a sphere (Vella et al., 2012b), an ellipsoid (Vella et al., 2012a), or a capped cylinder (Vogler et al., 2013; Weber et al., 2015), which helped the deduction of turgor pressure in various systems (Vogler et al., 2013; Beauzamy et al., 2015a,b; Weber et al., 2015).

Here we aim at generalizing shell models to tissues; accordingly, we do not make predictions at the sub-wall scale, but rather aim at providing a framework to guide indentation experiments at the cell scale. Our approach (Malgat et al., 2014) is based on the Simulation Open Framework Architecture (SOFA, Faure et al., 2012). We simulate the indentation of a floral meristem (Fernandez et al., 2010; Boudon et al., 2015) and vary turgor pressure and the elastic moduli of the different types of walls. We finally discuss our results in relation with recent experiments at cell scale (Peaucelle et al., 2011; Fernandes et al., 2012; Braybrook and Peaucelle, 2013; Milani et al., 2014; Beauzamy et al., 2015b; Peaucelle et al., 2015).

## 2. Materials and methods

In Malgat et al. (2014), we developed a mechanical model for the indentation of a plant tissue and we validated it for a square-shaped cell wall; we also illustrated the model with a template originating from a flower bud, assuming all cell walls to have the same elastic modulus. Here, we briefly recall the main modeling ingredients and explain the new ones.

### 2.1. Flower bud template

**Figure 1:**
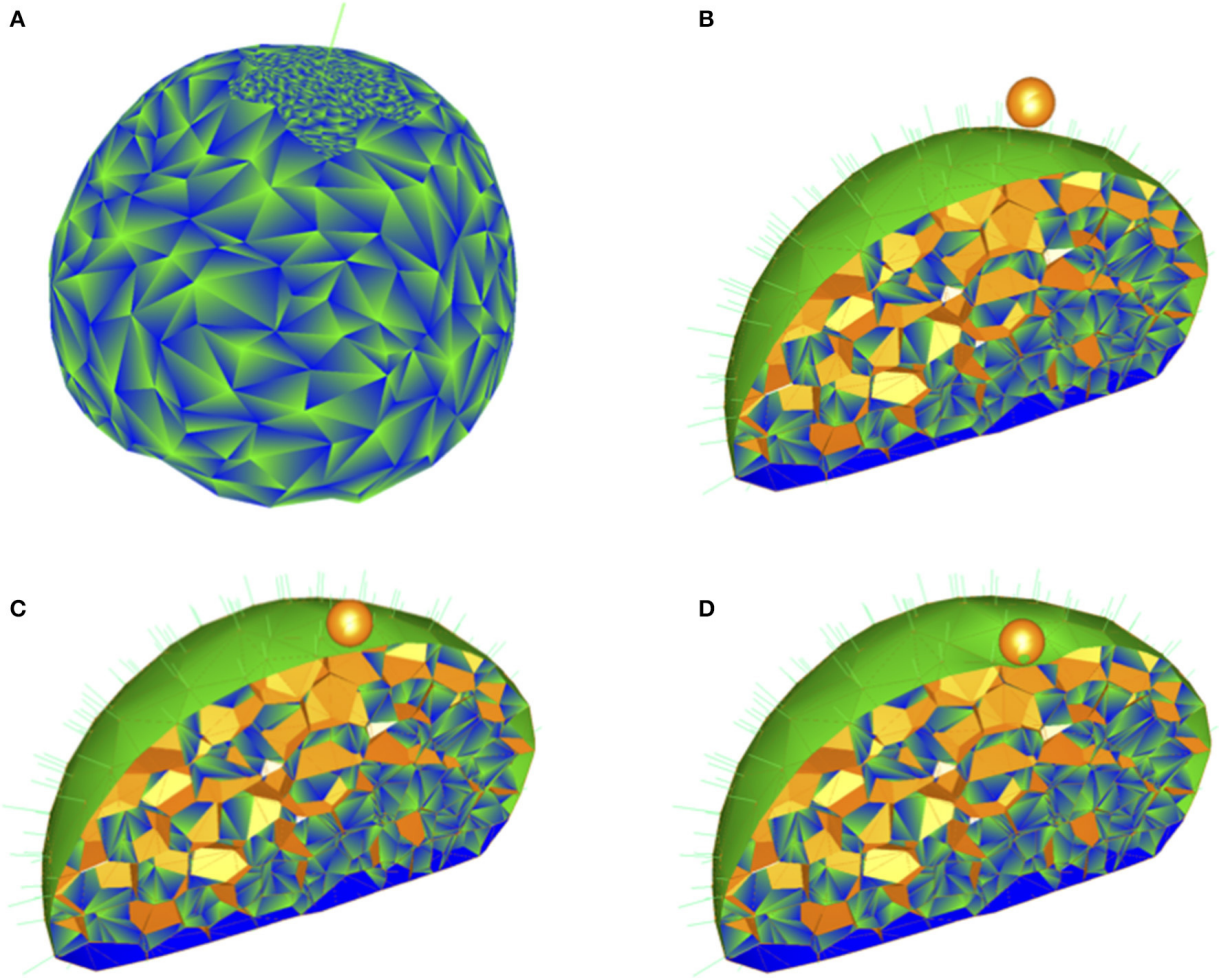
**Indentation of a three dimensional plant tissue by a spherical probe. (A)** Outer view of the triangular mesh, showing the refinement of the mesh at the indented cell and its neighbors. **(B–D)** Spherical probe and views of sectioned simulated flower bud before contact between probe and tissue **(B)**, at the first contact **(C)**, and at maximal displacement **(D)**. The colors were chosen to help visualization of facets and types of facets.

The template was obtained previously (Fernandez et al., 2010; Boudon et al., 2015). A flower bud was imaged by confocal microscopy and the 3D image was processed so as to extract the center of mass of each cell and the surface of the bud. The bud was partitioned into virtual cells using a Voronoi tesselation closed by the surface of the bud. The facets of cells were meshed with triangles and the mesh was refined near the probe in order to improve precision, see Figure 1A. For certain simulations, we defined categories of walls, based on the organization of the bud into cell layers: the L1 epidermal layer, the L2 subepidermal layer, and other cells (L3).

We also deformed this template by a small random displacement of all vertices and found force-displacement curves to be rather insensitive to such changes in the mesh (Supplementary Figure 1).

### 2.2. Constitutive elements of the model

Each cell wall (facet) was assumed to behave as a thin plate of thickness *h*, elastic modulus *E*, and Poisson ratio ν. Accordingly each wall has a stretching modulus *Eh* and a bending modulus *Eh*^3^/12(1−ν^2^). In addition to our previous work (Malgat et al., 2014), we also implemented anisotropy in the stretching modulus, which is modeled by the generalization of Hooke's law to an orthotropic material. Each thin plate is in plane stress, so that the stress tensor σ reduces to its 3 components in the (*x, y*) coordinates of the plate. Taking the notation ε for the strain tensor, Hooke's law can then be written as

$$\left[\begin{array}{c} \sigma_{\text{xx}}\\ \sigma_{\text{yy}}\\ \sigma_{\text{xy}} \end{array}\right]\;=\;\frac{1}{1-\nu_{\text{xy}}\nu_{\text{yx}}}\;\left[\begin{array}{ccc} E_{\text{x}} & \nu_{\text{yx}}E_{\text{x}} & 0\\ \nu_{\text{xy}}E_{\text{y}} & E_{\text{y}} & 0\\ 0 & 0 & G_{\text{xy}}(1-\nu_{\text{xy}}\nu_{\text{yx}}) \end{array}\right]\;\left[\begin{array}{c} \varepsilon_{\text{xx}}\\ \varepsilon_{\text{yy}}\\ 2\varepsilon_{\text{xy}} \end{array}\right]$$

with *E*_*i*_ the elastic modulus along axis *i*, *G*_*xy*_ the shear modulus in direction and ν_*ij*_ is the Poisson's ratio that corresponds to a contraction in direction *j* when an extension is applied in direction *i*. Symmetry gives the constraint ν_*xy*_/*E*_*x*_ = ν_*yx*_/*E*_*y*_. In the simulations with mechanical anisotropy, we varied the ratio α = *E*_*x*_/*E*_*y*_, keeping constant the following quantities: average modulus *E* = (*E*_*x*_+*E*_*y*_/)2, first Poisson's ratio ν_*xy*_ = ν, and the shear modulus *G*_*xy*_ = *E*/2(1+ν). Like other mechanical properties, mechanical anisotropy was assumed to be stationary. The direction of the stiffest direction was fixed based on qualitative observations of the cytoskeleton (see below).

The mechanical model was solved using the finite element method as implemented in the Simulation Open Framework Architecture (SOFA, Faure et al., 2012) and detailed in Malgat et al. (2014).

Also in addition to our previous work (Malgat et al., 2014), we considered a linear viscoelastic behavior, restricting ourselves to isotropic materials, whereby *E* is replaced by E+η d​/​dt. The corresponding viscoelastic time scale is τ = η/*E*. This constitutive law describes cell wall behavior at time scales that are small with respect to growth.

### 2.3. Physical parameters explored

The range of physical parameters is given in Table 1. Indentations were performed in the middle of a cell (around which the mesh was refined) and at its periphery (above an anticlinal wall). Except for the anisotropic cases, we show hereafter results obtained on a single cell from the top of the flower bud, though these results are typical of such locations, as our preliminary exploration revealed that the force-displacement curves did not vary qualitatively according to the exact location in the cell or to the cell probed.

**Table 1:** **Range of physical parameters**.

Typical cell size *R*	5μm
Modulus (*E*)	range 5–500 MPa
Poisson's ratio (ν)	0.49
Thickness (*h*)	0.4 μm
Mechanical anisotropy (α = *E*_*x*_/*E*_*y*_)	1:1 to 5:1
Pressure (*P*)	range 0.1–1 MPa
Probe diameter	0.6–5μm
Maximum displacement (δ)	10% of cell size or 0.5μm
Viscoelastic timescale (τ = η/*E*)	0.1–10 s
Time for force increase (*T*)	1 s

For each static force-displacement curve, equilibrium is computed for 30–50 successive values of the force (enough values to obtain a smooth curve), with force increments in the 10 nN range, using at each step a full implicit Euler solver coupled with a conjugate gradient solver (Malgat et al., 2014) until the convergence criterion is reached. As a consequence, each force-displacement curve requires a computation time ranging from 6 to 9 h on a standard desktop computer. The results presented here involve about a hundred such force-displacement curves.

In the case of the viscoelastic model, the force was increased according to the equation *F* = *F*_*m*_*t*/*T*, where *T* is the time needed to reach the maximal value *F*_*m*_, and then decreased according to *F* = *F*_*m*_(2−*t*/*T*). As we do not wait for equilibrium, simulations are significantly faster, yielding a force-displacement curve in less than an hour.

## 3. Results

### 3.1. Homogeneous stiffness of cell walls

**Figure 2:**
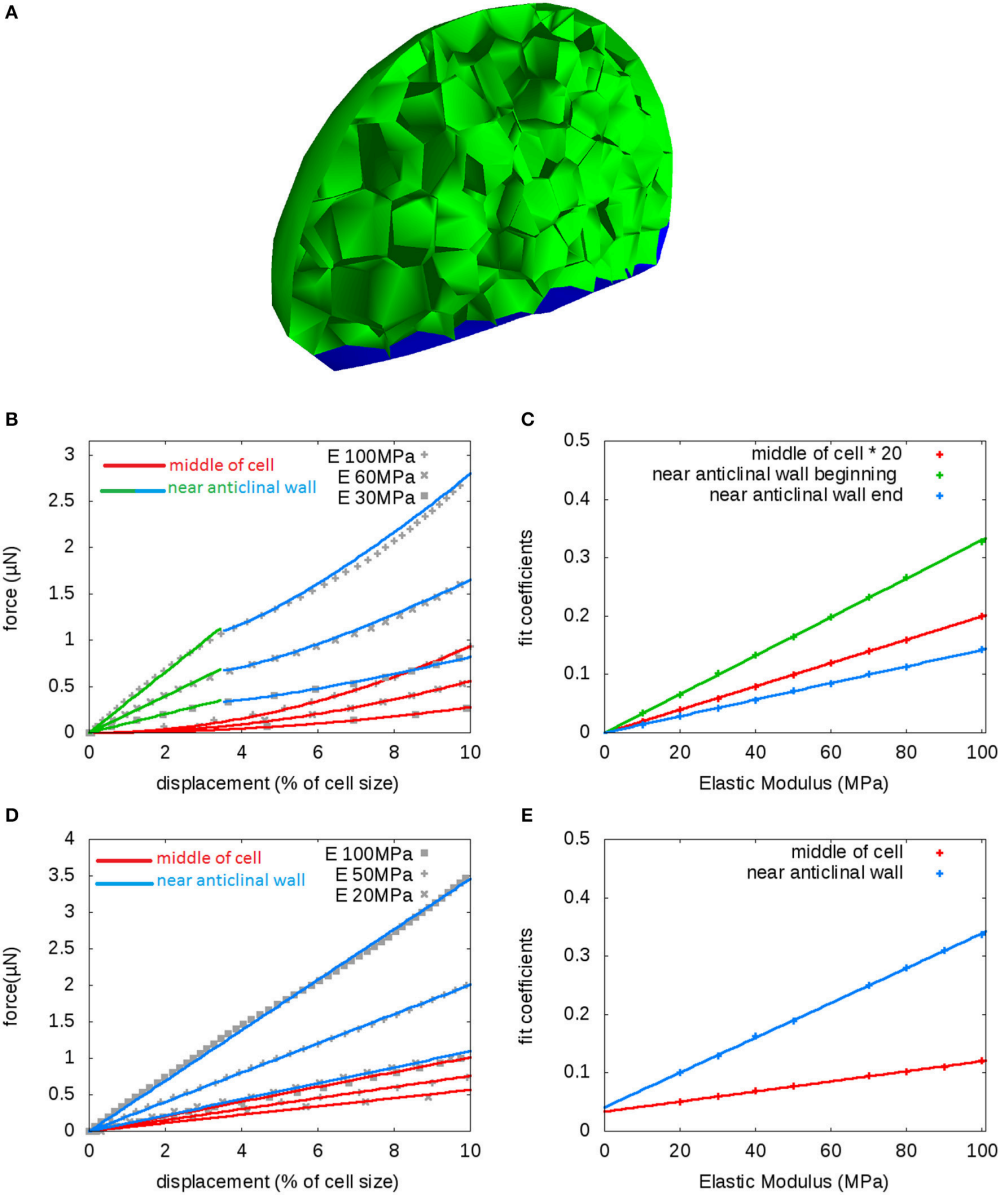
**Uniform elasticity: Indentation by a sphere (radius 5μm) of a flower bud with walls having uniform elastic modulus; sensitivity to modulus**. **(A)** Section showing fixed cell walls in blue. **(B–E)** Indentation with no turgor pressure **(B,C)** and with turgor *P* = 0.5 MPa **(D,E)**. **(B,D)** Force-displacement curves and power-law fits to the curves, near the cell middle and near an anticlinal wall, shown for three values of elastic modulus *E*. **(C,E)** Prefactors *a* of the fits to force-displacement curves as a function of the elastic modulus—note that in **(C)** the values for cell middle are multiplied by 20. With no pressure the exponent *b* takes the values 2 near the cell middle and 1 (small displacement) or 1.3 (large) near an anticlinal wall **(B,C)**, whereas with pressure the exponent *b* is always close to 1 consistently with the linear curves **(D,E)**.

We simulated the indentation of a flower bud by a spherical probe (Figures 1B–D) and we first assumed that all cell walls had the same elastic modulus (Figure 2A). We initially took a probe diameter of 5μm (Peaucelle et al., 2011; Braybrook and Peaucelle, 2013). In all cases, we found strain to be smaller than about 5%, consistent with our use of a linearly elastic material (Supplementary Figure 2).

We started with no pressure, as would be the case when tissues are plasmolyzed (Peaucelle et al., 2011; Braybrook and Peaucelle, 2013; Peaucelle et al., 2015). Force-displacement curves differ qualitatively according to whether indentation is performed near the cell middle or near an anticlinal cell wall (wall roughly perpendicular to the surface), as seen in Figure 2B. The vicinity of anticlinal cell walls appear effectively stiffer as about three times higher forces are needed to reach the same depth; the corresponding force-displacement, *F*(δ), curves exhibit two regimes, roughly linear at small displacement and superlinear (concave) at larger displacement. We fitted all curves to power-law equations with prefactor *a* and exponent *b*, *F* = *aδ*^*b*^, taking the origin of displacement at δ = 3.5% for the second regime at anticlinal walls. The exponents were *b* = 2 at cell middle and *b* = 1 (small depth) or *b* = 1.3 (large depth) at anticlinal walls. The superlinear curves are consistent with experimental observations (Peaucelle et al., 2011; Braybrook and Peaucelle, 2013). The linear behavior has not been observed, but it occurs at displacements smaller than wall thickness, which falls out of the scope of our model. All prefactors are proportional to the elastic modulus (Figure 2C), which yields the only force scale in the absence of turgor pressure.

We then switched to a pressurized state corresponding to experiments on turgid tissues (Milani et al., 2014; Beauzamy et al., 2015b) and took a reasonable value of pressure of *P* = 0.5 MPa. Curves are rather linear as can be seen in Figure 2D, whether indenting near an anticlinal wall or near the middle of a cell. We fitted these curves with linear equations, *F* = *aδ*, and found that the prefactor *a* depends linearly on the elastic modulus (Figure 2E), though this dependence is weak when indenting at the cell middle. Consequently, turgor pressure is the main parameter influencing indentation on periclinal walls, whereas cell wall and turgor contribute with comparable magnitude to these curves near an anticlinal wall. Altogether, it is the ratio of pressure to modulus, *P*/*E*, that matters for qualitative changes in behavior upon indentation.

**Figure 3:**
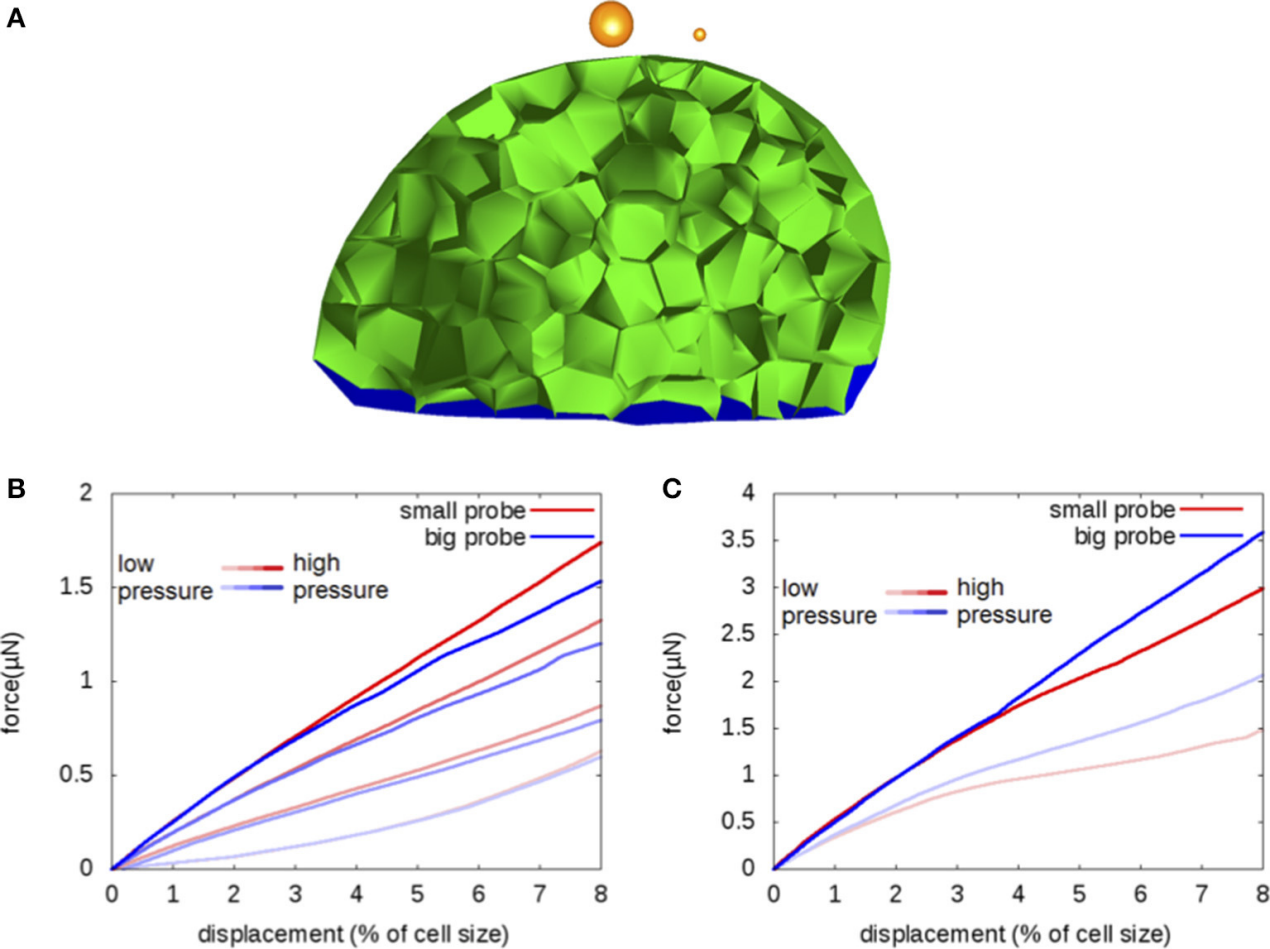
**Probe size: Indentation by a sphere of a flower bud with walls having uniform elastic modulus; sensitivity to probe size**. **(A)** Two probe sizes are considered: 0.6 and 5μm; the modulus is fixed at 50 MPa. **(B,C)** Force-displacement curves obtained near cell middle **(B)** or near anticlinal wall **(C)**. The values of pressure range from 0.1 MPa to 1 MPa; the lighter colors correspond to smaller values. The red and blue curves correspond to the small and large probe, respectively.

Finally, we studied the impact of probe size (Figure 3A) for different values of pressure, when indenting in the middle of a cell (Figure 3B) or near an anticlinal wall (Figure 3C). In all cases, probe size has little influence at small displacements and matters more at higher displacements. At the cell middle, a small probe yields higher forces than a large probe because the cell wall is locally more bent, as can be predicted in simple geometries (Vella et al., 2012b). In contrast, at the cell periphery, a small probe yields a smaller force: This might be explained by a smaller contact with the anticlinal wall, which has a larger mechanical resistance to indentation. At lower or zero pressure, probe size has a minimal influence at the cell's middle, whereas it has a relatively strong influence at cell periphery. Overall, probe size does not affect the qualitative behavior of force-displacement curves when pressure is varied.

### 3.2. Viscoelasticity

Experiments (Peaucelle et al., 2011; Fernandes et al., 2012; Braybrook and Peaucelle, 2013; Milani et al., 2014; Beauzamy et al., 2015b; Peaucelle et al., 2015) show hysteresis in force-displacement curves: approach (indentation) and retract (de-indentation) do not superimpose. We therefore explored the possibility of a linear viscoelastic behavior of the cell walls. We considered a Kelvin-Voigt constitutive law, where stress is the sum of an elastic stress that depends linearly on strain and of a viscous stress that depends linearly on strain rate. This constitutive law describes wall relaxation at time scales much smaller than the time scales of irreversible wall deformation associated with growth. The Kelvin-Voigt law yields a viscoelastic timescale τ = η/*E*, which is the ratio between viscosity η and elastic modulus *E*. The rate of force application becomes important when viscosity is accounted for. We considered that the force increases at constant rate up to its maximal value and then decreases to 0, the two phases having the same duration *T*. Dimensional analysis indicates that the results depend on the ratio, ρ = τ/*T*, of the two times.

**Figure 4:**
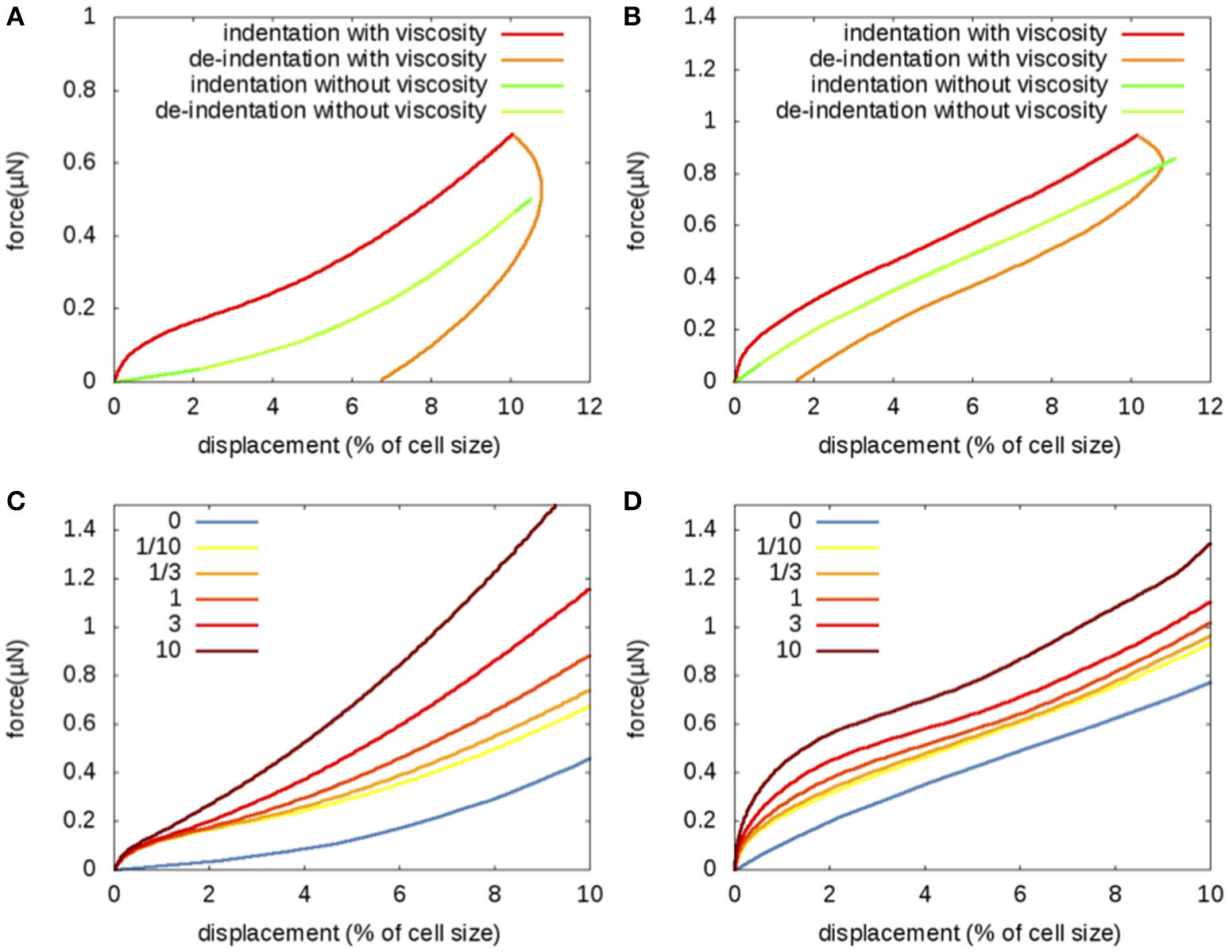
**Uniform viscoelasticity: Indentation by a sphere (radius 5μm) of a flower bud with viscoelastic walls having uniform elastic modulus ***E*** = 60 MPa and uniform viscosity η; sensitivity to the ratio ρ = τ/***T*** of the viscoelastic time scale, τ = η/***E***, to the duration of the force ramp, ***T***; same geometry as in Figure 2A**. **(A,B)** Approach and retract force-displacement curves for ρ = 1. **(C,D)** Approach curves for values of ρ = 0, 1/10, 1/3, 1, 3, and 10. **(A,C)** no pressure and **(B,D)** turgor pressure *P* = 0.5 MPa.

We first considered ρ = 1, and retrieved hysteresis in the force-displacement curves without (Figure 4A) and with (Figure 4B) turgor pressure, as in experiments; however unlike in experiments, indentation depth slightly increased after the force started to decrease, which might be ascribed to the sudden, unrealistic shift in force rate that we imposed. Hysteresis is smaller in a turgid bud, because pressure contributes significantly to apparent stiffness with no dependance on the velocity of loading. We then varied ρ from small to large values (Figures 4C,D). A higher viscosity always leads to a higher apparent stiffness upon approach. In experiments, the hysteresis is relatively small, suggesting that the viscous time scale is smaller than the force ramp duration and that wall viscosity has a smaller contribution than wall elasticity to tissue mechanical behavior.

### 3.3. Anisotropy

**Figure 5:**
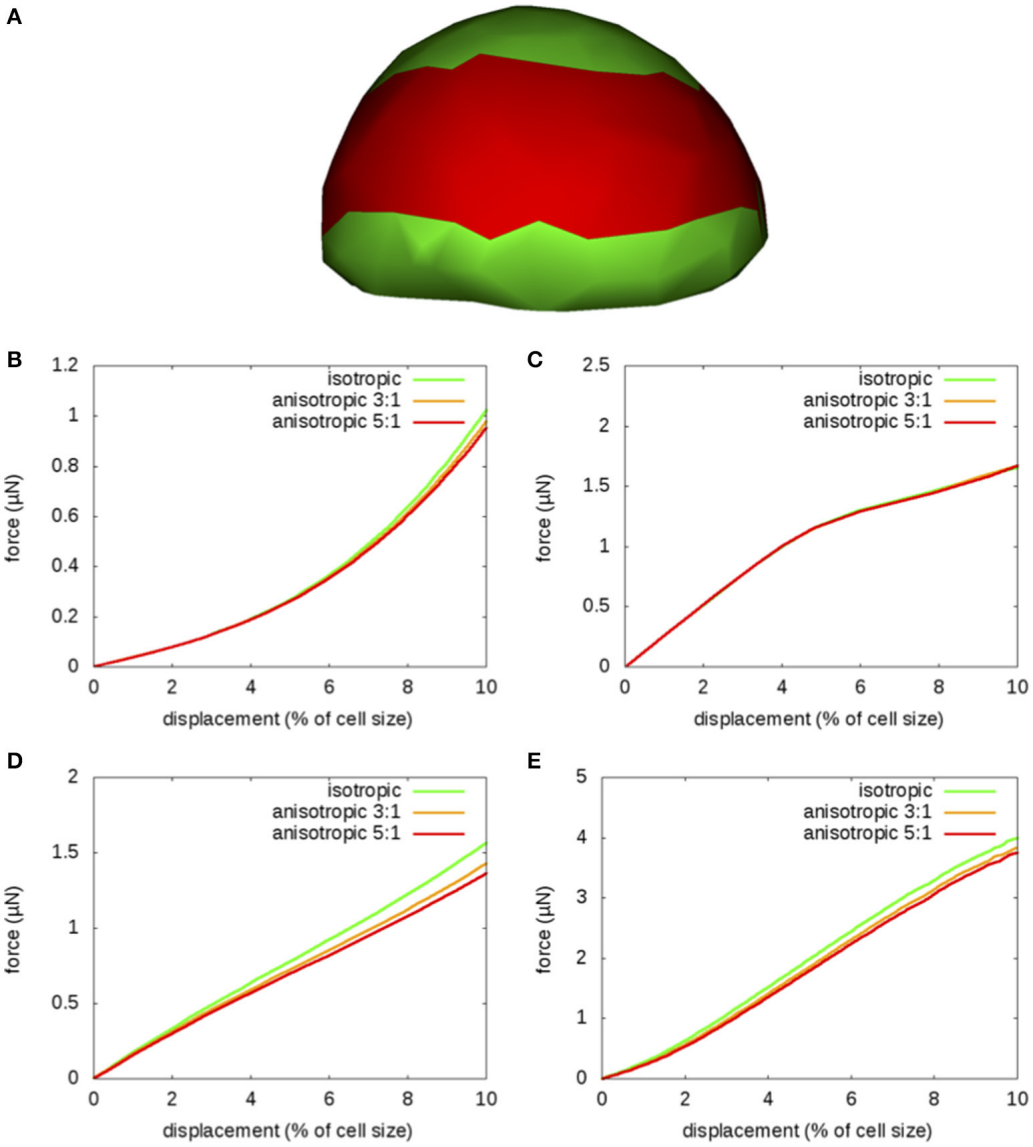
**Mechanical anisotropy: Indentation by a sphere of a flower bud with anisotropic surface walls; sensitivity to mechanical anisotropy**. **(A)** The flanks (red) are mechanically anisotropic; surface walls are stiffer in the circumferential direction (along the red band) and indentation is performed on a cell from the flanks. The radius of the probe is 5μm. **(B–E)** Force-displacement curves obtained with zero pressure **(B,C)** and a pressure *P* = 0.5 MPa **(D,E)**, near the cell middle **(B,D)** or above an anticlinal wall **(C,E)**. The anisotropy ratio takes the values 1:1 (isotropic), 3:1, and 5:1. The average elastic modulus is *E* = 100 MPa.

The cell wall can be mechanically anisotropic when cellulose fibrils are aligned (Cosgrove, 2015). For instance, the elastic modulus of epidermal onion peels is 5 times larger in the main direction of cellulose fibrils than in the perpendicular direction (Kerstens et al., 2001). Based on the orientation of cortical microtubules at the shoot apex (Hamant et al., 2008), surface walls are expected to be mechanically isotropic at the tip of the floral bud and anisotropic at the flanks with the stiffest direction circumferential to the tip. We simulated this configuration with the stiffest direction parallel to the red band shown in Figure 5A, and indented an anisotropic cell from this region, either at its middle or at its periphery. The cell indented had a refined mesh as in the isotropic case. The mean elastic modulus was kept constant, whereas the degree of anisotropy was varied from 1 (isotropic) to 5 (the elastic modulus in one direction is 5 times higher than in the perpendicular direction).

We first considered the case of no pressure. When indenting in the middle (Figure 5B), anisotropy has negligible influence on force-displacement curves at small depth (up to 6% displacement), and anisotropy makes the wall appear slightly softer (by about 5 %) at larger depths. When indenting at the cell periphery, anisotropy has no influence at all because anticlinal walls are dominant there (Figure 5C).

With pressure, the trends are similar to the plasmolyzed case, though they are somewhat amplified. The changes between the unpressurized case and the pressurized case might be ascribed to the small anisotropic inflation of cells. Mechanical anisotropy makes the wall appear softer (Figures 5D,E). The strongest effect of anisotropy occurs in the cell middle at larger depths: a 5:1 anisotropy makes cells appear softer by 15%.

Overall, mechanical anisotropy of the cell wall slightly reduces the apparent stiffness of the tissue.

### 3.4. Variations in stiffness of cell walls across layers

**Figure 6:**
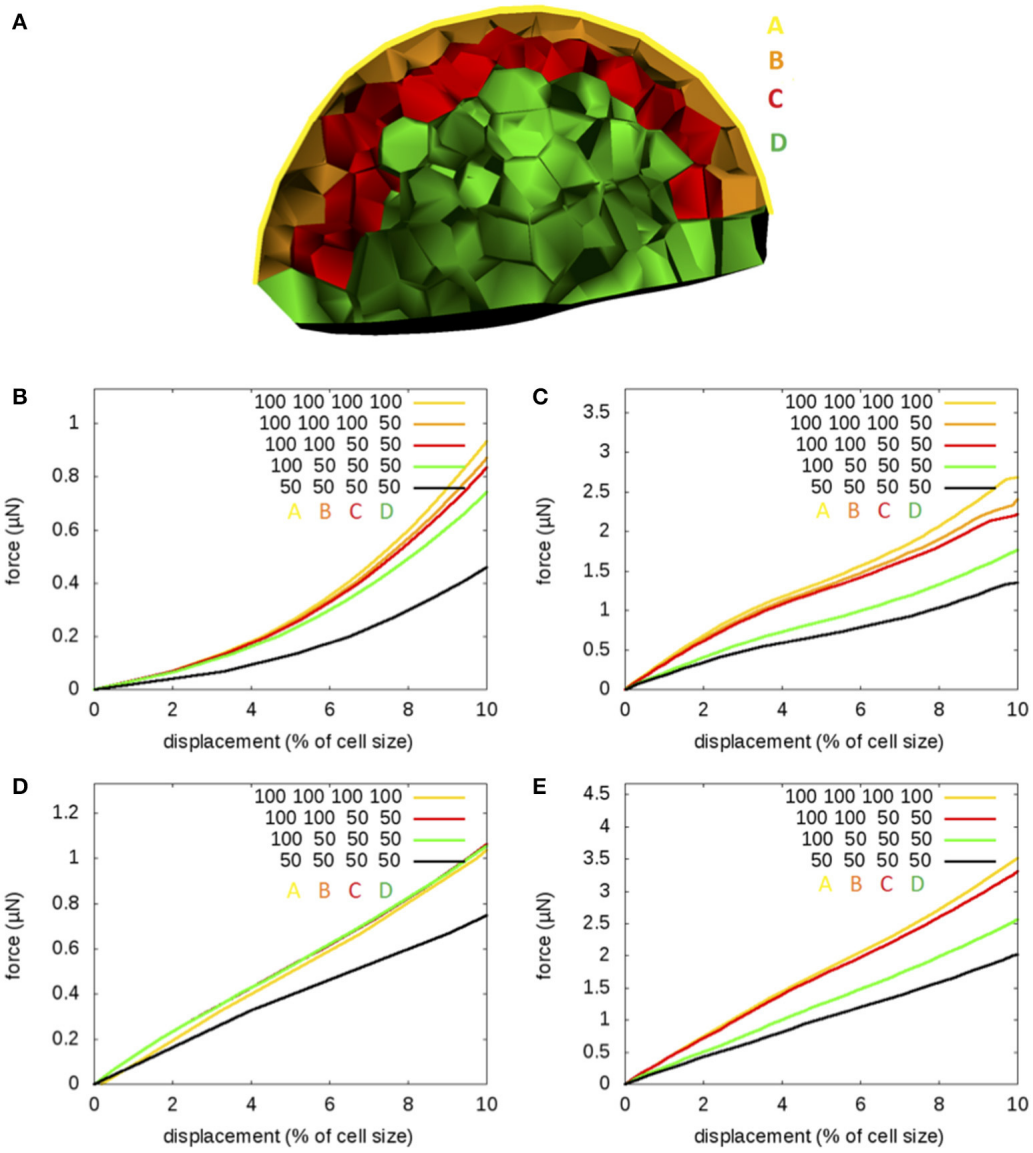
**Spatially varying elasticity: Indentation by a sphere of a flower bud where elastic modulus varies according to layers**. **(A)** Definition of the layers: A-surface walls, B-walls anticlinal to the surface, C-all walls of the second cell layer, and D-all other walls. **(B–E)** Force-displacement curves obtained with zero pressure **(B,C)** and a pressure *P* = 0.5 MPa **(D,E)**, near the cell middle **(B,D)** or above an anticlinal wall **(C,E)**. Elastic moduli (*E*_*A*_, *E*_*B*_, *E*_*C*_, *E*_*D*_) take the values (100, 100, 100, 100), (100, 100, 100, 50), (100, 100, 50, 50), (100, 50, 50, 50), or (50, 50, 50, 50) MPa, respectively.

Next, we released the assumption that cell wall stiffness is homogeneous, because there are many indications that, in the aerial part of the plant, wall stiffness decreases from the surface toward the inside (see e.g., Beauzamy et al., 2015b). We therefore subdivided cell walls in 4 categories (Figure 6A): surface walls (A, elastic modulus *E*_*A*_), anticlinal walls in the epidermal L1 cell layer (B, modulus *E*_*B*_), cell walls of the subepidermal L2 layer (C, modulus *E*_*C*_), and all other walls (D, modulus *E*_*D*_). We started with the common value 100 MPa for the elastic modulus and we gradually reduced the values in the different layers to finally get to a modulus that is half the initial value in all layers.

We first considered the case of no pressure. In the middle of the cell (Figure 6B), we observe that we need to reduce the modulus of anticlinal cell walls by a factor of 2 in order to reduce the resisting force by about 20%. Surface walls have a strong influence on force-displacement curves, whereas the L2 layer and deeper walls have little influence. Near the cell periphery (Figure 6C), the elastic modulus of anticlinal walls has the strongest influence on indentation curves, though surface walls have a comparable influence. D-walls do not contribute mechanically. Interestingly, reducing L2 cell walls by a factor of 2 can lead to a reduction of the resisting force by about 20%. This could be expected because the L2 is mechanically closer to anticlinal cell walls of the L1 than to the middle of surface cell walls.

In the pressurized case, indentation in the middle of the cell (Figure 6D) is only influenced by the elastic modulus of the cell wall, whereas indentation at the cell periphery (Figure 6E) is influenced by the moduli of both surface and anticlinal cell walls.

## 4. Discussion

Using a rather realistic mechanical model of a plant tissue indented by a spherical probe, we explored the behavior of force-displacement curves when varying probe size, contact location (near cell center or near anticlinal wall), elastic modulus of cell walls, viscosity of walls, mechanical anisotropy of walls, or turgor pressure. This mechanical model is directly relevant to many recent experimental studies (Peaucelle et al., 2011; Fernandes et al., 2012; Braybrook and Peaucelle, 2013; Milani et al., 2014; Beauzamy et al., 2015b; Peaucelle et al., 2015), though it does not apply to indentation depths significantly smaller than wall thickness (Milani et al., 2011; Hayot et al., 2012; Routier-Kierzkowska et al., 2012; Radotic et al., 2012; Forouzesh et al., 2013).

As a first approximation, indentation curves are mostly sensitive to the elastic modulus of surface walls and to turgor, while mechanical anisotropy has little influence on these curves. This is consistent with previous theoretical work in configurations corresponding to single cells (Vella et al., 2012a,b; Weber et al., 2015). Therefore the main parameter controlling the qualitative behavior of curves is the ratio of turgor to average elastic modulus, *P*/*E*, as well as the spatial variations in this ratio. If cell size, *R*, or wall thickness, *h*, were varied, work on thin shells (Vella et al., 2012a,b; Weber et al., 2015) suggests that *PR*/*Eh* should be the relevant parameter. The vicinity of anticlinal walls appears stiffer (Peaucelle et al., 2011; Braybrook and Peaucelle, 2013; Milani et al., 2014; Peaucelle et al., 2015), in the absence of other features such as a softer material above anticlinal cell walls in onion epidermis (Routier-Kierzkowska et al., 2012). We also find that a viscoelastic behavior of the cell walls may account for the hysteresis in force-displacement curves observed in experiments; however, with turgor pressure, such hysteresis might also originate in water movement in the tissue (Beauzamy et al., 2015b).

In turgid tissues, probe size does not affect the results qualitatively. Apparent stiffness in the cell middle is mostly sensitive to turgor, making it possible to infer turgor as performed by Beauzamy et al. (2015b). At the cell periphery, indentation is primarily sensitive to turgor and to the modulus of anticlinal walls and secondarily to surface walls. Milani et al. (2014) ascribed the difference in cell peripheral stiffness between the central zone and peripheral zone of aerial meristems to cell walls, though they did not exclude variations in turgor. This restriction is relevant based on the present results and future work should address whether there are spatial differences in turgor at the shoot apex.

In plasmolyzed tissues, probe size matters. With a small probe, apparent stiffness is mostly sensitive to walls of the epidermis - surface walls or anticlinal walls according to the location. With a probe comparable to cell size, apparent stiffness of anticlinal walls is also sensitive to the elastic modulus of the sub-epidermal L2 cell layer. This gives theoretical grounds to how Peaucelle et al. (2011) interpreted their results on the difference in stiffness between flower initia and meristem proper: finding no difference with a small probe and different values with a large probe, they concluded that cell walls of the L2 and L3 soften at initia. Our results indicate that this conclusion mostly holds for the L2.

An apparent limitation of our study is that we assumed that cell walls are linearly elastic, knowing that cell walls can exhibit nonlinear elasticity, with e.g., walls that are stiffer when under tension (Kierzkowski et al., 2012; Lipchinsky et al., 2013). Nevertheless, cell walls undergo only small deformations under indentation at the depths explored here. Given that any nonlinear material has an elastic modulus for each value of tension, the moduli considered in the present study for turgid tissues could be interpreted as the elastic moduli of the cell walls in the turgid state. Therefore, our work is broadly applicable to the interpretation of indentation experiments on plant tissues.

A last question is the relevance of our work to morphogenesis and more specifically to growth control. Strictly speaking, the expansion of cell walls is determined by their extensibility and not by their elasticity (Cosgrove, 2015). However, studies of mechanics of the shoot apex (as reviewed in Milani et al., 2013) suggest a correlation between elasticity and extensibility. In this context, our results can help constraining three-dimensional models of growing plant tissues (Boudon et al., 2015) that address the mechanical regulation of morphogenesis.

## Author contributions

RM, FF, and AB designed research; RM performed research; AB supervised the project.

## Funding

The work was funded by the INRIA Morphogenetics project, by the ANR (#12-BSV2-023-02), and by the ERC (Starting Grant Phymorph #307387).

### Conflict of interest statement

The authors declare that the research was conducted in the absence of any commercial or financial relationships that could be construed as a potential conflict of interest.
