# Supplementary material for: A Mechanical Model to Interpret Cell-Scale Indentation Experiments on Plant Tissues in Terms of Cell Wall Elasticity and Turgor Pressure
Source: Front Plant Sci. 2016 Sep 7;7:1351. doi: 10.3389/fpls.2016.01351 (PMC5013127; doi:10.3389/fpls.2016.01351)
Supplement: Supplementary file 1 [file Presentation1.pdf]

Supplementary Material A mechanical model to interpret cell-scale indentation experiments on plant tissues in terms of cell wall elasticity and turgor pressure

Richard Malgat, François Faure and Arezki Boudaoud
*Correspondence:
Arezki Boudaoud
arezki.boudaoud@ens-lyon.fr

CODE

The SOFA scripts for the indentation of a square are available at `https://forge.cbp.ens-lyon.fr/redmine/projects/afm-plane/`.

SUPPLEMENTARY FIGURES

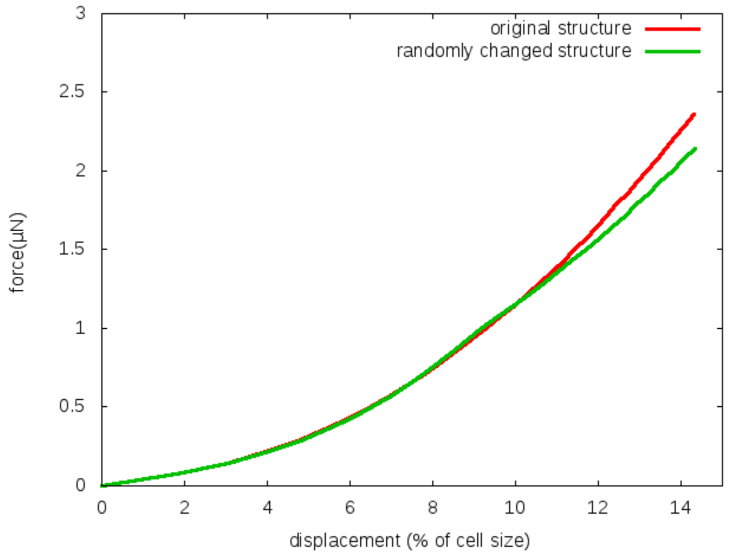

SUPPLEMENTARY FIGURE 1. Robustness with respect to geometry. Force-displacement curves obtained from indentation by a sphere (radius 5μm) of a flower bud with uniform elastic modulus ($E = 100$MPa). The original structure was deformed by displacing all vertices by a random vector with coordinates uniformly distributed in (-0.25μm,0.25μm), i.e. by about 5% of cell size.

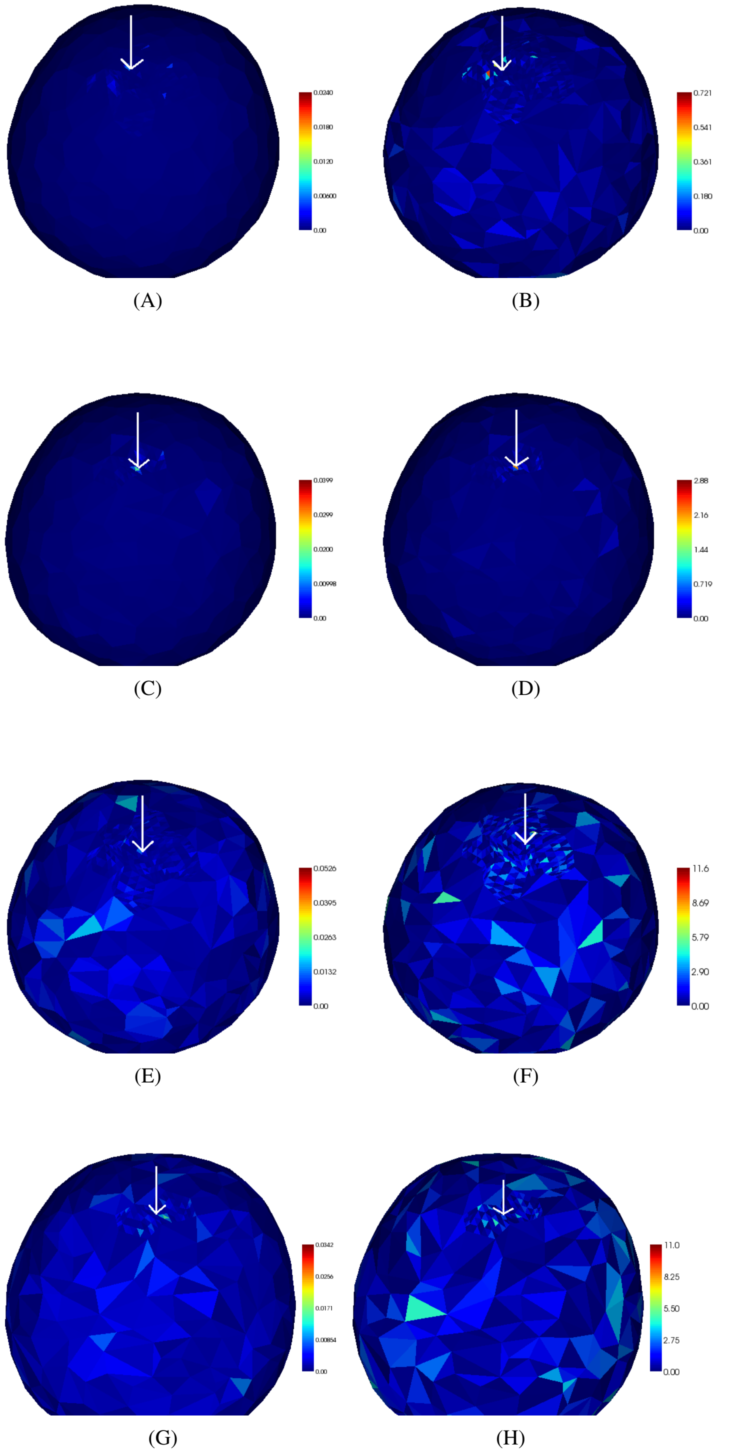

SUPPLEMENTARY FIGURE 2. Indentation by a sphere (radius 5μm) of a flower bud with uniform elastic modulus ($E = 60$MPa); strain (A,C,E,G) and stress (B,D,F,H), without (A-D) and with turgor pressure ($P = 0.5$MPa, E-H), at maximum indentation depth (10%); indentation near cell middle (A,B,E,F) and near anticlinal wall (C,D,G,H). The bar on the right of each plot shows the color scale of the norm (square root of the sum of squared components) of strain (unitless) or stress (in MPa).
